# A decade of antimicrobial resistance trends in *Pseudomonas aeruginosa*: insights from a tertiary care hospital in Saudi Arabia (2013–2022)

**DOI:** 10.3389/fmicb.2025.1617522

**Published:** 2025-07-07

**Authors:** Yahya Shabi, Abdullah Algarni, Ali Al bshabshe, Tarik Alazraqi, Glenn Patriquin, Abdulah OS Bawazeer, Safia Abdullah Mohammed, Sara Habbash, Ali Somily, Abdulah J. Alqahtani, Saeed M. S. Alhamhhum

**Affiliations:** ^1^Department of Microbiology and Clinical Parasitology, College of Medicine, King Khalid University, Abha, Saudi Arabia; ^2^Department of Family Medicine, Aseer Central Hospital, Abha, Saudi Arabia; ^3^Department of Medicine, College of Medicine, King Khalid University, Abha, Saudi Arabia; ^4^Department of Internal Medicine, Aseer Central Hospital, Abha, Saudi Arabia; ^5^Division of Microbiology, Department of Pathology and Laboratory Medicine, Nova Scotia Health, Halifax, NSW, Canada; ^6^Department of Microbiology, Aseer Central Hospital, Abha, Saudi Arabia; ^7^Unit of Microbiology, Department of Pathology, King Khalid University Hospital, Riyadh, Saudi Arabia

**Keywords:** *Pseudomonas aeruginosa*, nosocomial infections, multidrug-resistant (MDR), extensively drug-resistant (XDR), difficult-to-treat resistance (DTR), Saudi Arabia

## Abstract

**Background:**

*Pseudomonas aeruginosa* (PA) is a significant cause of nosocomial infections, and increasing antimicrobial resistance complicates management.

**Objectives:**

To characterize antimicrobial susceptibility trends, we conducted a retrospective study of PA clinical isolates over 10 years (2013–2022) at a tertiary care hospital in Saudi Arabia.

**Results:**

A total of 2,490 PA isolates were analyzed (1,452 from general wards and 1,038 from ICUs). Carbapenem resistance was observed in 40% of isolates; 37.5% were multidrug-resistant (MDR), 5.3% were extensively drug-resistant (XDR), and 3.5% met the criteria for difficult-to-treat resistance (DTR).

**Conclusion:**

This study provides an overview of PA resistance pattern trends in Saudi Arabia and emphasizes the importance of establishing an antimicrobial stewardship program.

## Introduction

*Pseudomonas aeruginosa* (PA) is an opportunistic, non-glucose-fermenting, aerobic Gram-negative bacillus in the Pseudomonadaceae family. It is ubiquitous in moist environments. PA rarely causes community-acquired infections and more commonly causes healthcare-associated infections. Notably, PA accounts for approximately 7.1–7.3% of all healthcare-associated infections (Reynolds and Kollef, 2021). It is particularly problematic in hospitals since it can contaminate medical equipment and cause nosocomial outbreaks (Banerjee and Stableforth, 2000). Patients in intensive care units (ICUs) are at especially high risk, with ~16.2% of all ICU isolates reported to be PA. The most common site of PA infection is the lower respiratory tract, especially among mechanically ventilated patients, due to biofilm formation in endotracheal tubes leading to ventilator-associated pneumonia (Reynolds and Kollef, 2021; Banerjee and Stableforth, 2000; Nadeem et al., 2009). These infections carry high morbidity and mortality (32–42%). PA can also cause urinary tract infections in catheterized patients (it is isolated in ~16% of all catheter-associated UTIs in ICUs) and surgical site infections in ~4–6% of cases (Reynolds and Kollef, 2021). Other possible infection sites include the skin (especially in burn patients) and the bloodstream, often with significant morbidity and mortality (Pruitt et al., 1998; Ranjan et al., 2010; Micek et al., 2005). Certain patient populations, such as those who are neutropenic or have cystic fibrosis, are particularly at risk for severe PA infections. Mechanically ventilated patients are at high risk due to the organism’s ability to form biofilm inside tubes and introduce it to the lungs through aspiration. PA can infect surgical sites in 4.3–5.6% of cases (Ranjan et al., 2010). Special populations at risk include neutropenic patients and those with cystic fibrosis.

PA possesses multiple intrinsic resistance mechanisms that render it non-susceptible to many antibiotics. It is intrinsically resistant to drugs such as ampicillin, amoxicillin (with or without *β*-lactamase inhibitors), first-and second-generation cephalosporins, ertapenem, tetracycline, tigecycline, trimethoprim-sulfamethoxazole, and chloramphenicol. Recently Clinical and Laboratory Standards Institute (CLSI) guidelines, gentamicin breakpoints for PA were removed due to high intrinsic resistance [Clinical and Laboratory Standards Institute (CLSI), 2023]. Key intrinsic resistance mechanisms of PA include a chromosomally encoded AmpC *β*-lactamase (which inactivates many β-lactams) and multidrug efflux pumps that expel various antibiotics (β-lactams, chloramphenicol, fluoroquinolones, macrolides, sulfonamides, tetracyclines, trimethoprim, and aminoglycosides). Due to efficient efflux and limited porin uptake, PA is also intrinsically less susceptible to ertapenem. These intrinsic mechanisms typically do not confer resistance to certain potent anti-pseudomonal agents, such as piperacillin, ceftazidime, cefepime, ceftolozane-tazobactam, meropenem, imipenem, ciprofloxacin, levofloxacin, tobramycin, amikacin, or colistin. However, PA can develop adaptive resistance to these agents during exposure via AmpC hyperproduction or efflux pump upregulation. PA also acquires resistance through genetic mutations (e.g., porin loss or regulatory mutations) and horizontal gene transfer (e.g., plasmid-borne *β*-lactamases).

Infections with such highly resistant isolates are extremely challenging to treat and are associated with increased patient morbidity and mortality (Centers for Disease Control and Prevention (CDC), 2019). Poor infection control practices and inappropriate antimicrobial use (e.g., overuse of broad-spectrum empiric therapy) have contributed to rising resistance trends, a problem exacerbated during the COVID-19 pandemic (Abubakar et al., 2023; Aldawsari et al., 2020). There are limited contemporary studies from this region Saudi Arabia characterizing PA resistance patterns [World Health Organization (WHO), 2017]. Therefore, we conducted this 10-year retrospective analysis to identify and characterize the antimicrobial susceptibility trends of PA isolates from nosocomial infections in a tertiary care hospital in Saudi Arabia, covering the period 2013–2022.

## Materials and methods

### Study design and setting

We performed a retrospective 10-year analysis at a 500-bed tertiary care hospital in Saudi Arabia, examining the antimicrobial resistance patterns of PA from January 2013 through December 2022.

### Data collection

All non-duplicate clinical isolates of PA from hospital-associated infections during the study period were included. Isolates were obtained from both general ward and ICU patients, encompassing a variety of specimen sources (e.g., respiratory tract samples, urine, blood, wound swabs, abscesses, body fluids, cerebrospinal fluid, and soft tissue). Only the first isolate per patient infection episode was included. Clinical specimens were collected using sterile techniques as per hospital protocol and transported to the microbiology laboratory for immediate processing.

### Microbiological analysis

Samples underwent Gram staining and were inoculated onto appropriate culture media (sheep blood agar, chocolate agar, MacConkey agar, and cysteine lactose electrolyte-deficient agar) according to standard laboratory procedures (Jorgensen et al., 2019). Culture plates were incubated at 35–37°C and examined for bacterial growth at 24 and 48 h. Suspected PA colonies (based on characteristic morphology and oxidase positivity) were identified at the species level using an automated identification system (VITEK 2, bioMérieux, USA). Antimicrobial susceptibility testing was performed using VITEK 2 automated methods, and resistant results were confirmed by the manual methods using Mueller-Hinton agar (Oxoid, UK) when necessary, following CLSI guidelines. The panel of tested antipseudomonal antibiotics was selected according to annual CLSI recommendations. Susceptibility results were interpreted using CLSI breakpoints (Colistin susceptibility was not categorized as “Susceptible” per CLSI M100 guidelines, which only define intermediate or resistant breakpoints for PA).

### Data analysis

Isolate data, including source, hospital location (ICU vs. ward), and antibiotic susceptibility results, were recorded in Microsoft Excel. Categorical data (e.g., proportion of resistant vs. susceptible isolates by year, ward, or specimen source) were analyzed using Fisher’s exact test. Trends in resistance over time were evaluated using a binomial generalized linear model. A *p*-value < 0.05 was considered statistically significant. Analyses were performed using SPSS version 20 (IBM Corp., Armonk, NY, USA).

### Ethical consideration

The data collection was done after obtaining official permission from Ministry of Health, Aseer branch review board (F7-2-2025).

## Results

### Patient demographics

A total of 2,490 PA isolates were included in this study, including 1,452 from general wards and 1,038 from ICU. There was an overall decline in PA isolates from 446 in 2012/2013–192 in 2022. Overall, prevalence among male patients was greater than among females, comprising 68.5% of the total number of isolates between 2013 and 2022 (Table 1). The source of the isolate and resistance profiles throughout the study period are represented in Table 1. PA was mostly isolated from respiratory samples (45.2%), wound samples (20.1%), and urine (19.9%), followed by blood (5.1%). Other samples included skin/soft tissues, bone, eye samples, aspirated fluids, and CSF (Table 1).

**Table 1:** Characteristics of the study population and resistance profiles.

Characteristics	Total (*N* = 2,490)	CRPA (*n* = 997)	MDR (*n* = 934)	XDR (*n* = 132)	DTR (*n* = 87)	*p*-value
Sex, no. (%)						
Male	1,706 (68.5)	697 (40.9)	636 (37.3)	78 (4.6)	58 (3.4)	0.235
Female	784 (31.5)	300 (38.3)	298 (38.0)	54 (6.9)	29 (3.7)	
Hospital location, no. (%)						<0.001
General Wards	1,452 (58.3)	505 (34.8)	452 (31.1)	66 (4.5)	41 (2.8)	
ICU	1,038 (41.7)	492 (47.4)	482 (46.4)	66 (6.4)	46 (4.4)	

Culture source	CRPA rate	MDR rate	XDR rate	DTR rate	Key findings
Respiratory (*n* = 1,125)	50.1%	46.6%	6.0%	4.2%	Highest CRPA/MDR rates (likely due to biofilm formation in ventilated patients).
Wound (*n* = 500)	28.8%	28.4%	4.2%	3.2%	Lower resistance vs. respiratory (*p* < 0.001 for CRPA/MDR).
Urine (*n* = 496)	34.9%	33.7%	5.8%	2.4%	Intermediate resistance; XDR rate similar to respiratory (*p* = 0.87).
Blood (*n* = 128)	36.7%	35.9%	6.3%	5.5%	Highest DTR rate (*p* = 0.03 vs. other sources).
Other (*n* = 241)	28.6%	22.8%	2.9%	2.1%	Lowest resistance rates overall (*p* < 0.001 for all vs. respiratory).

Year	Total isolates	CRPA rate (%)	MDR rate (%)	XDR rate (%)	DTR rate (%)	Key trends
2013	446	28.9	48.9	4.0	0.9	Baseline year; highest MDR (48.9%, *p* < 0.001 vs. 2014–2017).
2014	316	34.5	32.9	0.6	0.0	Significant drop in MDR/XDR (*p* < 0.001).
2015	253	40.7	35.6	0.4	0.0	Rising CRPA (*p* = 0.02 vs. 2013).
2016	274	45.6	36.1	0.7	0.0	Peak CRPA (45.6%, *p* < 0.001 vs. 2013).
2017	227	43.2	33.0	1.3	0.0	Stabilized resistance.
2018	201	43.8	32.3	11.4	8.0	XDR/DTR spikes (*p* < 0.001 vs. prior years).
2019	251	32.7	29.9	7.2	8.0	High DTR persists (*p* = 0.002).
2020	139	40.3	41.0	14.4	13.7	Highest XDR/DTR (COVID-19 impact; *p* < 0.001).
2021	191	50.3	37.7	16.2	14.7	CRPA/DTR peaks (*p* < 0.001).
2022	192	57.8	41.1	7.3	0.0	CRPA surges (57.8%, *p* < 0.001), but XDR/DTR drop (*p* = 0.03).

### Antimicrobial resistance profile

Clinically, PA isolates are often classified by resistance phenotypes. In this study, we categorized isolates as follows: Carbapenem-resistant *P. aeruginosa* (CRPA) – resistant to at least one carbapenem (imipenem or meropenem); multidrug-resistant *P. aeruginosa* (MDR-PA) – non-susceptible to at least one agent in three or more antimicrobial classes; extensively drug-resistant *P. aeruginosa* (XDR-PA) – non-susceptible to all but two or fewer antimicrobial classes; and difficult-to-treat resistance (DTR) – non-susceptibility to all first-line anti-pseudomonal agents, including piperacillin-tazobactam, ceftazidime, cefepime, aztreonam, meropenem, imipenem-cilastatin, ciprofloxacin, and levofloxacin (Magiorakos et al., 2012; Kadri et al., 2018).

Among the 2,490 PA isolates, 997 (40.0%) met the definition of carbapenem-resistant PA (CRPA), 934 (37.5%) were classified as MDR-PA, 132 (5.3%) were XDR-PA, and 87 (3.5%) were DTR-PA. These resistance phenotype categories were more frequently observed in ICU isolates compared to ward isolates. In particular, CRPA, MDR-PA, and DTR-PA proportions were significantly higher among ICU-derived isolates than those from general wards (*p* < 0.05 for each comparison in Table 1). By contrast, the distribution of these phenotypes did not differ significantly by patient sex.

**Figure 1:**
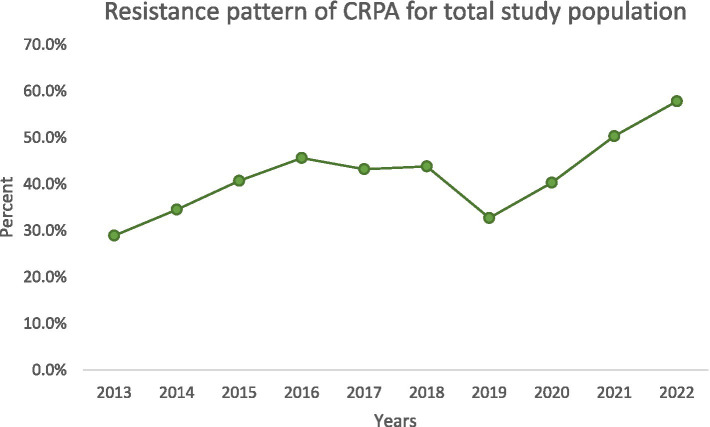
Trend of CRPA incidence.

Overall susceptibility rates for key antipseudomonal agents during the study period are as follows. The aminoglycosides were the most active: amikacin had the highest susceptibility rate at 76%, and tobramycin was 75% susceptible. Fluoroquinolone susceptibilities were 67% for levofloxacin and 65% for ciprofloxacin. Among *β*-lactams, piperacillin-tazobactam retained 65% susceptibility, while ceftazidime and cefepime were each 64% susceptible. For carbapenems, 64% of isolates were susceptible to meropenem and 58% were susceptible to imipenem. Aztreonam had the lowest susceptibility rate at only 54% (Colistin results are not characterized as “susceptible” per CLSI criteria and thus are not included in the susceptibility percentages). These cumulative susceptibility data are illustrated in the composite antibiogram (Figure 1).

### Trends in resistance over time

**Figure 2:**
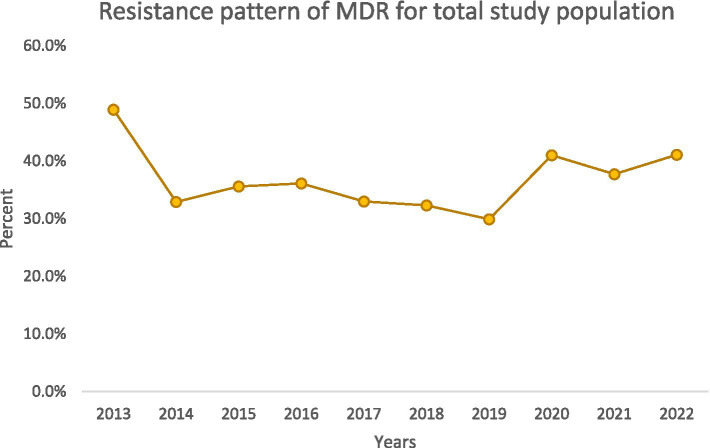
Trend of MDR-PA incidence.

**Figure 3:**
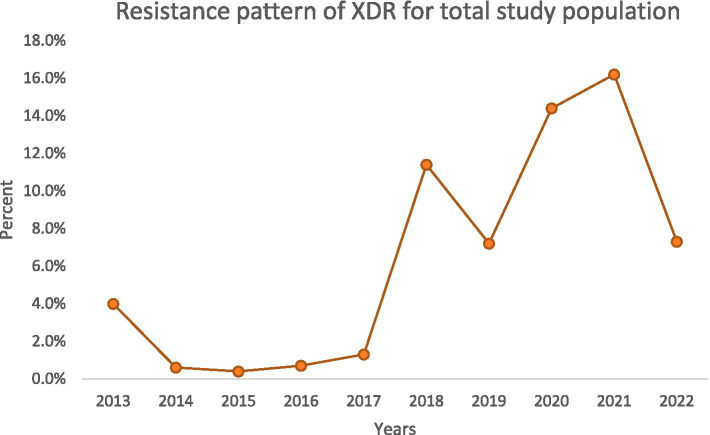
Trend of XDR-PA incidence.

**Figure 4:**
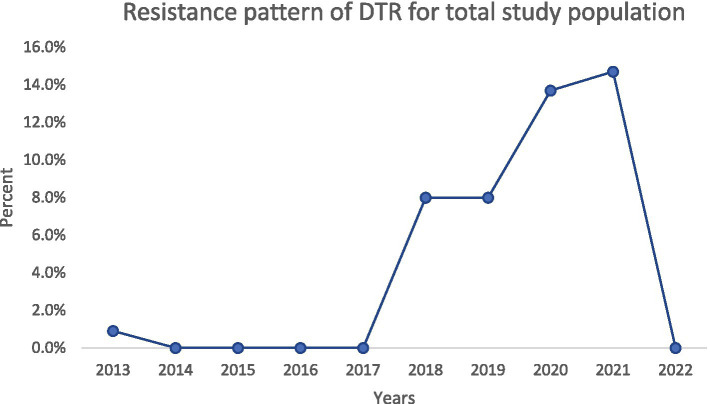
Trend of DTR-PA incidence.

Over the decade, we noted important temporal trends (Figures 1–4). The incidence of CRPA showed fluctuation but an overall increase in the middle years of the study before a slight decline toward 2021–2022 (Figure 1). A similar pattern was observed for MDR-PA (Figure 2). XDR-PA remained infrequent each year (generally <5% of annual isolates), though a slight uptick was seen in the latter half of the study (Figure 3). DTR-PA isolates were rare throughout, with annual proportions mostly under 2%, except for isolated spikes (Figure 4).

**Figure 5:**
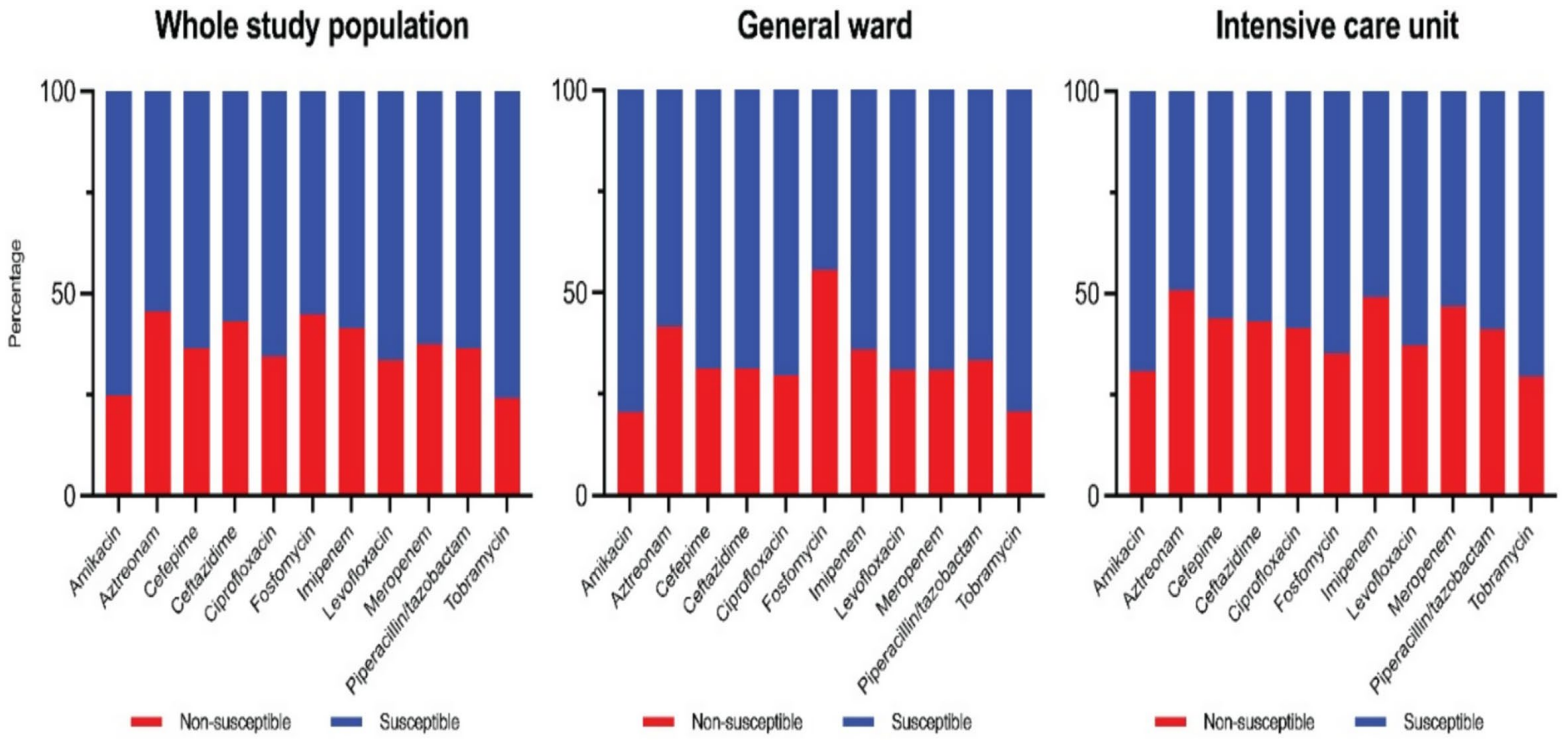
Antibiogram of PA over 10-year period from all isolates.

The compiled antibiogram of PA over the 10 years (Figure 5) illustrates the relative consistency of susceptibilities for most drugs, with a slight downward trend in susceptibility to several agents over time. Notably, carbapenem susceptibility declined over the early years and stabilized after 2018 (reflecting the trends in CRPA rates). Fluoroquinolone susceptibility showed a gradual decrease corresponding to increased use during the COVID-19 pandemic years.

## Discussion

PA is a major cause of hospital-acquired infections, and its multiple intrinsic and acquired resistance mechanisms often limit treatment options. Effective empiric therapy for suspected PA infections is challenging, underscoring local resistance surveillance’s importance in guiding clinicians. In recognition of the threat, the World Health Organization (WHO) in 2017 categorized PA as a Priority 1 (“critical”) pathogen on the list of antibiotic-resistant bacteria in urgent need of new therapies (Ahmed, 2016). In this context, our study provides a longitudinal analysis of PA resistance patterns over 10 years in a Saudi tertiary care hospital.

Encouragingly, we observed a substantial decline in the total number of PA isolates recovered annually, from 446 in 2013 to 192 in 2022. This reduction could reflect improved infection control measures, hospital admission case mix changes, or effects of the COVID-19 pandemic (during which elective procedures were reduced). However, despite fewer isolates, the proportion of drug-resistant phenotypes increased in both ICU and general ward settings over time. We documented high resistance rates in key categories: overall, 40% of isolates were CRPA, 37.5% were MDR-PA, 5.3% were XDR-PA, and 3.5% were DTR-PA. Notably, CRPA, MDR-PA, and DTR-PA were significantly more common among ICU isolates than ward isolates (*p* < 0.05), highlighting the greater selective pressure and vulnerable patient population in the ICU. These findings align with global reports that ICU settings tend to harbor more resistant PA strains due to intensive antibiotic usage and prolonged patient exposures.

Our resistance rates for specific antipseudomonal agents are comparable to some reports from other centers in Saudi Arabia (Momenah et al., 2023). For example, we observed resistance in approximately one-third of isolates to piperacillin-tazobactam (35%), ceftazidime (36%), and cefepime (36%), which is similar to rates documented in a prior Saudi hospital study (39.8, 32.4, and 36.1%, respectively). However, our meropenem and ciprofloxacin resistance rates (37 and 35%) were higher than those reported in that study (28.6 and 22.2%) (Alatoom et al., 2024). These discrepancies could be due to local differences in antibiotic prescribing practices and infection control. In our hospital, widespread empiric use of carbapenems and fluoroquinolones—especially during the early phase of the COVID-19 pandemic—may have driven the higher resistance observed to meropenem and ciprofloxacin. Inadequate antimicrobial stewardship (e.g., prolonged empiric therapy without de-escalation) and lapses in infection control can facilitate the emergence and spread of resistant PA, particularly in ICU settings. Indeed, increased use of broad-spectrum antibiotics and failure to promptly de-escalate therapy in critically ill patients (including those with COVID-19) likely contributed to the resistance trends we observed. Strengthening antibiotic stewardship interventions and ensuring compliance (such as obtaining cultures before starting therapy and de-escalating based on sensitivities) are critical steps to mitigate this issue.

Regarding infection sources, our study found that respiratory tract infections constituted the largest proportion of PA cases (>45%), followed by wound and urine sources. This contrasts with a recent 11-year study from Makkah (western Saudi Arabia) in which wound infections were the most common source of PA (39.6%), followed by respiratory specimens (31.2%) and then urine (8.7%). Bloodstream infection rates were low in both studies (around 5%) [World Health Organization (WHO), 2017]. The higher respiratory isolation rate in our center may reflect a focus on ventilated ICU patients and pneumonia cases, whereas the Makkah hospital might have seen more post-surgical wound infections. Such differences underscore the importance of considering local patient demographics and hospital specialties when comparing infection epidemiology.

We also noted a male predominance in PA infections (roughly 69% male vs. 31% female patients), consistent with other reports that have observed higher rates of PA infection in males. The reason for this gender difference is not fully clear but could relate to higher exposure risk in male patients or underlying comorbidities. In our data, the male predominance did not reach statistical significance (*p* = 0.23), suggesting that sex by itself was not a strong predictor of infection likelihood or resistance profile.

### Limitations

This study is limited by its retrospective design and reliance on laboratory records. We did not perform molecular typing or genetic analyses of the PA isolates; thus, we cannot delineate the specific resistance mechanisms (e.g., the presence of carbapenemase genes) underlying the phenotypes observed. Additionally, we lacked detailed clinical data on patient outcomes, preventing analysis of the impact of resistant infections on mortality, length of hospital stays, or treatment success. Despite these limitations, the large sample size and decade-long span provide a robust overview of resistance trends. Future studies should incorporate molecular characterization and clinical outcome correlations to build on these findings.

## Conclusion

This 10-year study offers a comprehensive overview of PA antimicrobial resistance patterns in a tertiary care hospital in Saudi Arabia. We documented high MDR and CRPA rates and notable occurrences of XDR and DTR isolates, particularly in ICU settings. Our findings provide valuable local data to inform clinicians and policymakers in designing targeted infection control strategies and optimizing antimicrobial stewardship programs. Ongoing surveillance and preventive interventions are essential to contain PA resistance and improve clinical outcomes in infected patients.

## Data Availability

The original contributions presented in the study are included in the article/supplementary material, further inquiries can be directed to the corresponding author.

## References

[ref1] Abubakar U. Al-Anazi M. Alanazi Z. Rodríguez-Baño J. (2023). Impact of COVID-19 pandemic on multidrug-resistant gram-positive and gram-negative pathogens: a systematic review. J. Infect. Public Health 16, 320–331. doi: 10.1016/j.jiph.2022.12.022, PMID: 36657243 PMC9804969

[ref2] Ahmed O. B. (2016). Incidence and antibiotic susceptibility pattern of *Pseudomonas aeruginosa* isolated from inpatients in two tertiary hospitals. Clin Microbiol 5, 2–4.

[ref3] Alatoom A. Alattas M. Alraddadi B. Moubareck C. A. Hassanien A. Jamal W.. (2024). Antimicrobial resistance profiles of *Pseudomonas aeruginosa* in the Arabian gulf region over a 12-year period (2010-2021). J. Epidemiol. Glob. Health. 14, 529–548. doi: 10.1007/s44197-024-00191-y, PMID: 38856819 PMC11442796

[ref4] Aldawsari A. Tawfik K. Al-Zaagi I. Sr. (2020). Antimicrobial-resistant bacteria and prescription of antibiotics at a tertiary care hospital in Riyadh, Saudi Arabia. Cureus 12:e12098. doi: 10.7759/cureus.12098, PMID: 33489515 PMC7805534

[ref5] Banerjee D. Stableforth D. (2000). The treatment of respiratory pseudomonas infection in cystic fibrosis: what drug and which way? Drugs 60, 1053–1064. doi: 10.2165/00003495-200060050-00006, PMID: 11129122

[ref6] Centers for Disease Control and Prevention (CDC) (2019). Antibiotic resistance threats in the United States, 2019. Atlanta, GA: U.S. Department of Health and Human Services, CDC.

[ref7] Clinical and Laboratory Standards Institute (CLSI) (2023). Performance standards for antimicrobial susceptibility testing, 33rd ed. Wayne, PA: CLSI.

[ref8] Jorgensen J. H. Pfaller M. A. Carroll K. C. Funke G. Landry M. L. Richter S. S.. (2019). Manual of clinical microbiology. 12th Edn. Washington, DC: ASM Press.

[ref9] Kadri S. S. Adjemian J. Lai Y. L. Spaulding A. B. Ricotta E. Prevots D. R.. (2018). Difficult-to-treat resistance in gram-negative bacteremia at 173 US hospitals: retrospective cohort analysis of prevalence, predictors, and outcome of resistance to all first-line agents. Clin. Infect. Dis. 67, 1803–1814. doi: 10.1093/cid/ciy378, PMID: 30052813 PMC6260171

[ref10] Magiorakos A. P. Srinivasan A. Carey R. B. Carmeli Y. Falagas M. E. Giske C. G.. (2012). Multidrug-resistant, extensively drug-resistant and pandrug-resistant bacteria: an international expert proposal for interim standard definitions for acquired resistance. Clin. Microbiol. Infect. 18, 268–281. doi: 10.1111/j.1469-0691.2011.03570.x, PMID: 21793988

[ref11] Micek S. T. Lloyd A. E. Ritchie D. J. Reichley R. M. Fraser V. J. Kollef M. H. (2005). *Pseudomonas aeruginosa* bloodstream infection: importance of appropriate initial antimicrobial treatment. Antimicrob. Agents Chemother. 49, 1306–1311. doi: 10.1128/AAC.49.4.1306-1311.2005, PMID: 15793102 PMC1068618

[ref12] Momenah A. M. Bakri R. A. Jalal N. A. Ashgar S. S. Felemban R. F. Bantun F.. (2023). Antimicrobial resistance pattern of *Pseudomonas aeruginosa*: an 11-year experience in a tertiary care hospital in Makkah, Saudi Arabia. Infect Drug Resist. 16, 4113–4122. doi: 10.2147/IDR.S409726, PMID: 37396063 PMC10312329

[ref13] Nadeem S. G. Qasmi S. A. Afaque F. Saleem M. Hakim S. T. (2009). Comparison of the in vitro susceptibility of clinical isolates of *Pseudomonas aeruginosa* in a local hospital setting in Karachi, Pakistan. Br J Med Pract 2, 35–39.

[ref14] Pruitt B. A. Jr. McManus A. T. Kim S. H. Goodwin C. W. (1998). Burn wound infections: current status. World J. Surg. 22, 135–145. doi: 10.1007/s002689900361, PMID: 9451928

[ref15] Ranjan K. P. Ranjan N. Bansal S. K. Arora D. R. (2010). Prevalence of *Pseudomonas aeruginosa* in post-operative wound infection in a referral hospital in Haryana, India. J. Lab Phys. 2, 74–77. doi: 10.4103/0974-2727.72153, PMID: 21346900 PMC3040092

[ref16] Reynolds D. Kollef M. (2021). The epidemiology, pathogenesis, and treatment of *Pseudomonas aeruginosa* infections: an update. Drugs 81, 2117–2131. doi: 10.1007/s40265-021-01635-6, PMID: 34743315 PMC8572145

[ref17] World Health Organization (WHO) (2017). WHO publishes list of bacteria for which new antibiotics are urgently needed. (news release, 27 February 2017). Geneva: World Health Organization.

